# Psychometric validation of the Vaccination Attitudes Examination (VAX) scale in German pre-pandemic and mid-pandemic samples

**DOI:** 10.1038/s41598-024-82726-8

**Published:** 2024-12-18

**Authors:** Marcel Wilhelm, Friederike L. Bender, Frank Euteneuer, Stefan Salzmann, Anne-Catherine I. Ewen, Winfried Rief

**Affiliations:** 1 Division of Clinical Psychology and Psychotherapy, Department of Psychology, Philipps-University of Marburg, Gutenbergstraße 18, 35032 Marburg, Germany; 2 Faculty of Human Sciences, Department of Psychology, Vinzenz Pallotti University, Vallendar, Germany; 3 Medical Psychology, Health and Medical University Erfurt, Erfurt, Germany

**Keywords:** Vaccine hesitancy, Vaccination behavior, Vaccination attitudes, Vaccine, VAX scale, German version, Psychology, Health care

## Abstract

Despite the public health value of immunizations, vaccine hesitancy is a widespread phenomenon and received special attention during the global COVID-19 pandemic. The Vaccination Attitudes Examination (VAX) Scale aims to identify anti-vaccination attitudes for understanding vaccine hesitancy. The present study is the validation of the German version of the VAX scale in pre-/mid-pandemic samples. All individuals completed an online-survey assessing prior and expected future vaccination behaviors, further constructs associated with vaccine hesitancy, as well as anti-vaccination attitudes using the translated VAX scale among others. In a first study, 297 individuals were surveyed before the COVID-19 pandemic. A second study included 260 individuals recruited during the COVID-19 pandemic. For validation, two confirmatory factor analyses have been conducted. Reliability measures (internal consistency, test–retest-correlation) were determined. The four subscales were confirmed by Confirmation Factor Analysis with a very good to excellent fit. The subscales showed good to excellent reliability and convergent validity determinants. Average VAX values significantly distinguished between individuals who had previously received or refused immunization and predicted expected vaccination behavior. The German translation of the VAX scale is effective in identifying anti-vaccination attitudes and can be used for further research on anti-vaccination attitudes and vaccine hesitancy.

**Supplementary Information:**

The online version contains supplementary material available at 10.1038/s41598-024-82726-8.

## Introduction

The COVID-19 pandemic has been a litmus test of the importance of public trust in immunization to overcome this public health threat^1^Germany, in particular, had one of the highest proportions of people who were unvaccinated and unwilling to get vaccinated in Europe amid the pandemic (02/15/2022: 19%; (Imperial College London, 2020–2022)). At the same time (02/14/2022), Germany listed 1,193.271 confirmed COVID-19 cases (World Health Organization, 2020–2023). However, such patterns of immunization rates and decline of vaccinations are consistently observable all over the world^2^. The reluctance or delay in acceptance of vaccinations when services applying them are available and accessible, has been defined as *vaccine hesitancy*^3^*.*In 2019, before the COVID-19 pandemic emerged, the World Health Organization (WHO) already listed the problem of vaccine hesitancy as one of the top ten health threats^4^. The current study aims to validate a German translation of the Vaccination Attitudes Examination (VAX) Scale in pre-pandemic and mid-pandemic samples to assess its psychometric properties and suitability for measuring anti-vaccination attitudes in German-speaking populations.

Definitions of vaccine hesitancy stem from psychological theories for predicting prevention behavior and comprise determinants such as confidence, complacency, or convenience/constraints^3,5^. Vaccine hesitancy is described as “complex and context specific, varying across time, place and vaccines”^3^. Consequently, many existing instruments assessing behavior and attitudes associated with vaccine hesitancy, focus on specific vaccines (e.g., Carolina HPV Immunization Attitudes and Beliefs Scale^6^) or frequently target parental populations (e.g., Vaccination confidence scale^7^). Other earlier scales target additional factors such as calculation referring to an individual’s effort in searching for comprehensive information or the willingness for collective responsibility (such as the 5C scale^5^). While these other scales adopt a theory- and literature-based structure to capture psychological antecedents of vaccination, the VAX Scale’s inductive development approach offers complementary insights. By deriving items from real-world public statements in diverse contexts, such as physician waiting rooms and anti-vaccine blogs, the VAX Scale captures nuanced and authentic dimensions of vaccine hesitancy. This community-grounded approach reveals aspects of anti-vaccination sentiment that may not emerge from theory-driven frameworks like the 5C. Thus, the VAX Scale serves as a valuable tool in contexts where understanding general vaccine hesitancy is prioritized, while the 5C may be more suitable for exploring psychological drivers of vaccine behavior.

In contrast to these existing questionnaires, Martin and Petrie^8^developed a 12-item questionnaire to assess the different facets of general anti-vaccination attitudes. This approach appears useful for the purpose of understanding vaccination campaign failures across different vaccines^9^and does still allow evolving vaccine-related concern-tailored interventions^10^. As their scale has already been translated into other languages ​​such as Italian and Spanish^11,12^, it appears to be a promising tool to measure vaccination attitudes across national borders. What distinguishes the Vaccination Attitudes Examination (VAX) Scale from other instruments that assess attitudes towards vaccination (e.g., Sarathchandra et al.^13^) is that the initial item pool was not only derived from thorough literature research but also created by qualitative approaches. While other scales, such as the 5C, are widely used and grounded in theoretical models, the VAX Scale’s inductive approach offers a distinct advantage. Derived directly from public statements in real-world contexts—such as physician waiting rooms and anti-vaccine blogs—the VAX Scale captures authentic dimensions of vaccine hesitancy that may be overlooked by theory-driven models. This allows the VAX Scale to address unique attitudinal aspects, such as mistrust in vaccine motives and preference for natural immunity, providing insights particularly relevant to general anti-vaccination sentiment across diverse vaccines, beyond context-specific hesitancy^14^.

Four specific subscales were found in an U.S. population^8^and replicated in UK residents^15^: (a) mistrust of vaccine benefit, (b) worries about unforeseen future effects, (c) concerns about commercial profiteering, and (d) preference for natural immunity. These four factors reflect concerns and perceived benefits of vaccinations as incorporated into an implicit cost–benefit analysis according to the necessity concern framework^8,16^. Concerns about medications and beliefs about the necessity of medication are closely related to medication adherence^16^and vaccination uptake^17^and show strong associations with perceived sensitivity to medicine, which is another indicator of a person’s concern to be particularly sensitive to medications^16^. Accordingly, higher average VAX values were associated with stronger anti-vaccination attitudes and were found to differentiate vaccinated individuals from non-vaccinated individuals^8^. After measles outbreaks, vaccination myths or mistrust to health authorities are often discussed as the main causes for low vaccination uptake in the public debate^14^. Since individual risk perception influences vaccination decisions and behavior, it seems logical to integrate such concerns into a questionnaire to determine a valid and reliable construct of vaccination attitudes^18^. In line with this assumption, the 5C scale^5^, for example, was supplemented by two factors during the course of the COVID-19 pandemic in order to continue to be able to accurately assess vaccination readiness: compliance or the support for societal monitoring to control adherence to regulations and conspiracy, which refers to the tendency to agree with conspiratorial beliefs^19^. The COVID-19 pandemic has once again confirmed that vaccination can protect against severe courses of diseases and thus against overloading the healthcare system^20^. From this public health perspective, it can be helpful to be able to map stable anti-vaccination attitudes over time to be able to draw conclusions from non-pandemic times to pandemic times and to derive consequences.

The aim of the present study was to develop a German translation of the Vaccination Attitudes Examination (VAX) Scale and to validate it in a non-pandemic and a pandemic German sample. For this reason (1) latent factor structure was tested in both samples; (2) internal consistency and retest-reliability estimates were evaluated; (3) construct validity was determined by assessing associations between average VAX values and conceptually related constructs; and (4) VAX scores were applied to differentiate between vaccinated individuals and non-vaccinated individuals.

According to prior findings^8,15^ (1) a replication of the 4-factor-structure was assumed by conducting two confirmatory factor analyses (sample 1 and 2). (2) VAX values were suggested to demonstrate high internal consistency and test–retest correlations (study 1 and 2). (3) High VAX scores were proposed to be positively associated with perceived sensitivity to medicine, negative beliefs about medicine, modern health worries, conspiracy mentality, online media consumption behavior, and the sub-factors complacency, calculation, and constraints of the 5C psychological antecedents of vaccination behavior scale. Negative associations were postulated for the 5C subscales confidence and collective responsibility (study 1 and 2). (4) In addition, high VAX values were supposed to distinguish between past influenza vaccination behavior and future willingness to get vaccinated against influenza and a pandemic Ebola, avian flu or COVID-19 virus (study 1 and 2). While this study evaluates the psychometric qualities of the VAX Scale, it does not include a comprehensive comparison with other established vaccine hesitancy scales, such as the 5C Scale. This distinction is made to focus on the validation of the VAX Scale within German-speaking contexts.

## Methods

### Design and procedure

Ethical approval was obtained by the Ethics Committee of the Department of Psychology, of the University of Marburg (reference number 2019-65 k) prior to the start of both studies. Informed consent was obtained from all individual participants included in the study prior to their participation. All procedures performed with human participants were in accordance with the ethical standards as laid down in the 1964 Declaration of Helsinki and its later amendments or comparable ethical standards.

**Fig. 1:**
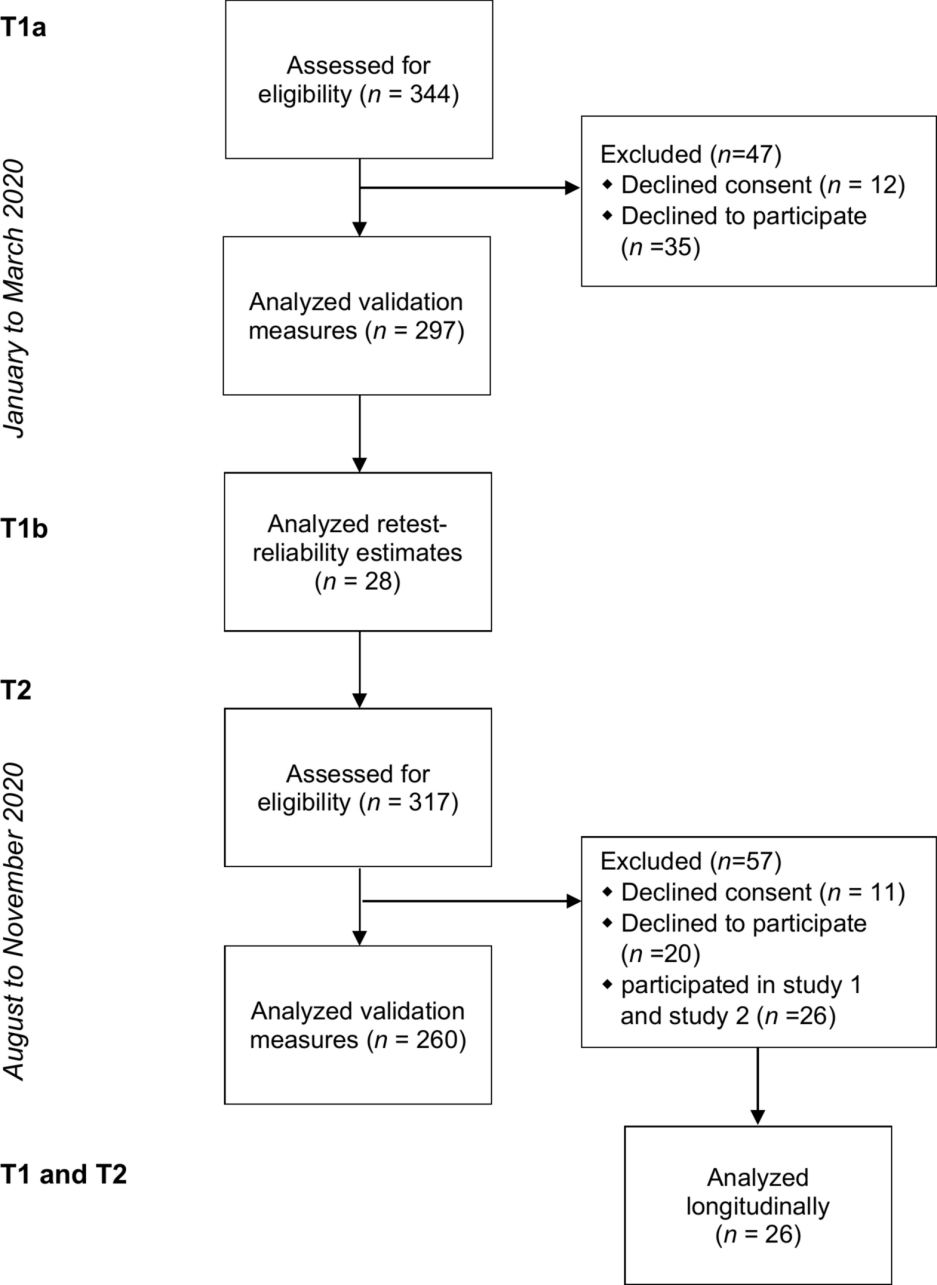
Flow diagram representing trial profile and participants.

Two survey periods with a total of three times of measurement (Fig. 1) were conducted. The first survey phase started on 13 January 2020 (T1a) and included a second measurement date four weeks later assessing retest-reliability estimates (T1b). It lasted until 23 March 2020, just before a first lockdown began in Germany (03/22/2020), which was associated with numerous restrictions in public life. Until then, an initial increase in the number of infections was recorded, but was limited to a few local areas. A second survey phase commenced on 27 August and lasted until 27 November 2020 (T2). After a significant decrease in the number of infections was recorded over the summer months, a second wave of infections began at this time. Schools started again in Germany after the summer break, mandatory masks and tests have been discussed in some federal states, and the first test phases of the corona vaccines began. Germany experienced the first demonstrations against the federal government’s corona policy, e.g., by vaccine opponents. Those participants who had already taken part in the first survey were excluded from the sample of the second survey phase (T1-T2).

The online self-report questionnaire was presented via SoSci Survey^21^ and the data was pseudonymized using a code. First, demographics were listed, followed by the Vaccination Attitude Examination (VAX) Scale, and the further validation items (see measures).

### Participants and recruitment

Eligible to participate in the study were all persons older than 18 years and able to understand the questionnaires in German. An a priori power analysis was conducted to determine the required sample size for the confirmatory factor analysis (CFA) using the RMSEA as the effect measure (semPower package in R). The results indicated that a sample size of 249 participants is required to achieve the desired power level in each sample. Another a priori power analysis resulted in a required sample size of 29 participants (1-ß = 0.80, α < 0.05, ρ = 0.48) for test–retest reliability estimates and 90 respondents (1-ß = 0.80, α < 0.05, ρ = 0.41) for longitudinal analysis of validation measures (T1-T2) taking into account effect sizes determined by Martin & Petrie^8^. Incomplete datasets were excluded from the analysis.

Participant acquisition took place via the university’s internal research participation system, mailing lists, and social networks (i.e., facebook, twitter). As compensation for participation, a voucher raffle was offered, which could be redeemed in various supermarkets. Six vouchers of €25 could be gained. Respondents who participated in both study 1 and study 2 were able to take part in a raffle for a €50-voucher. In total, 344 participants followed the link, agreed the informed consent and generated a code in study 1. Of these, 297 completed the study and 28 respondents were included in the sample assessing the retest reliability estimates after four weeks. In study 2, of 317 prospective participants 286 completed the survey. Of these participants, 26 participants could be identified as participants in study 1 and were included in a longitudinal analysis, even though the target sample size was not reached (see Fig. 1). Thus, the final data set of study 2 resulted in a sample size of 260.

### Measures

#### Vaccination Attitude Examination Scale

The English version of the VAX scale was translated into German following the guidelines of Beaton et al.^22^. With the help of two German native speakers, two German translations were created and merged into one, which was back-translated by two English native speakers naïve to the construct. The back translation was reviewed by a member of Keith Petrie’s working group, Faculty of Medical and Health Sciences, Psychological Medicine, University of Auckland, New Zealand. A pretest with 13 German experts (researchers and clinical psychologists) checked the translated version (using free text items) for linguistic plausibility and potential other meanings of the items. As a result, 70% of the final items were assigned to the intended subscale and, on average, 90% of the testers found the final items linguistically plausible. As in the English version, respondents rated their level of agreement with each item, three of which were reverse-coded (e.g., “I feel safe after being vaccinated”), on a six-point Likert scale ranging from 1 “strongly disagree” to 6 “strongly agree”.

#### Convergent and discriminant validity measures

Perceived sensitivity to medicine (PSM), which describes the perceptions of susceptibility to potential beneficial and adverse medication effects was assessed using the PSM scale developed by Horne, Faasse, et al.^23^. Five items are presented as statements (e.g., “My body overreacts to medicines”). All items have a five-point Likert answer option (ranging from 1 “strongly disagree” to 5 “strongly agree”) with responses adding up to sum scores ranging from 5 to 25. Higher scores reflect higher perceived sensitivity to medicine and were associated with increased reported symptoms and medication non-adherence^23^.

The Beliefs About Medicine Questionnaire (BMQ) by Horne et al.^24^assessed the cognitive representation in terms of beliefs and worries about medication. For the validation purpose of the present study, only the eight-item BMQ-General section was used, which comprises two subscales (General-Harm and General-Overuse) and assesses beliefs about medication in general. Respondents were asked to rate their level of agreement with each item (e.g., “Doctors use too many medicines”) on a five-point Likert-type scale (from 1 “strongly disagree” to 5 “strongly agree”). Higher values indicate more negative beliefs and have been strongly associated with the aversion of prescribed and over-the-counter medicines and the use of herbal remedies^25^. Significantly higher BMQ-overuse scores were found in chronically ill patients who had refused a vaccine compared to those who had not^17^.

Modern Health Worries (MHW) were recorded by the translated version of the MHW Scale by Bailer et al.^26^which assessed individuals’ concerns about aspects of modern life (e.g., overuse of antibiotics, traffic fumes, additives in food, radio or cell phone towers) on a five-point Likert-type scale (ranging from 1 “not concerned” to 5 “extremely concerned”). A 25-item version of the scale was used. Individuals with many and varied modern health worries use less prescribed medication^27^. and tended to be more interested in alternative health care products^28^.

The five-item Conspiracy Mentality Questionnaire (CMQ^29^) was used to assess the general tendency to believe in theories that view complex world events and crises as a result of elaborate plots devised by secret and powerful forces. An eleven-point Likert-type scale (ranging from 0 “0%, certainly not” to “100%, certain”) was used to assess the likelihood of items being true (e.g., “I think that government agencies closely monitor all citizens”). Previous studies have found an association between conspiracy mentality and anti-vaccination attitudes^5^.

The long-version, 15-item Psychological Antecedents of Vaccination Behavior (5C) Scale was developed by Betsch et al^5^. to record relevant predictors of vaccination behavior. In contrast to the VAX scale, its item conception is based on theoretical frameworks such as those of the Strategic Advisory Group of Experts on Immunization (SAGE) advising the WHO^3^. In total, the scale comprises five factors: complacency (a perceived low risk of vaccine-preventable disease), calculation (individuals’ engagement in information seeking), and constraints (referring to barriers in the availability and accessibility of vaccinations and the willingness to overcome them), which were hypothesized to be associated with anti-vaccination attitudes^3^. Confidence (in vaccine’s effectiveness and safety) and collective responsibility (the willingness to protect others) were assumed as discriminatory measures of validity^5,30^. Items were presented with seven-point Likert answer categories ranging from 1 “strongly disagree” to 7 “strongly agree”.

Online media consumption behavior reflects a preference to research health topics online and had previously shown associations with anti-vaccination attitudes^8^It was assessed using a six-point Likert-type scale (from 1 “never” to 6 “always”)^8^.

#### Criterion validation measures

To quantify criterion validity, prior vaccination behavior and vaccination intentions were recorded according to the method of^8^. To differentiate between vaccinated individuals and non-vaccinated individuals two dichotomous (yes/no) items asked participants whether they had received an influenza vaccination in the past flu season, and whether they intended to get vaccinated next season. To inquire about vaccination intentions during a pandemic, participants were asked to rate the likelihood of being vaccinated against Ebola or avian influenza in the event of an outbreak and an available vaccination on a six-point Likert scale (from 1 “very unlikely” to 6 “very likely”). In study 2, this item was supplemented by the intention to receive a COVID-19 vaccination.

### Statistical analyses

Analyses were conducted using RStudio version 1.2.5042. First, the VAX scale values were subjected to a multivariate outlier analysis considering the leverage values according to Stevens^31^. In study 1, four outliers were identified, in study 2, no outliers were found. The authors decided to include the outliers in the calculations to depict a representative sample. A comprehensive item-analysis was calculated with the first and second sample, including item difficulty, item-total correlations for item discrimination, and Cronbach’s alpha as reliability measure. The lavaan package^32^ was used to calculate the confirmatory factor analyses (CFA).

The factor analyses were followed by pairwise correlations, *t*-tests and linear regression models to evaluate test–retest reliability and associations between the average VAX scale score and the values of the Perceived Sensitivity to Medicine Scale, the Beliefs about Medicine Questionnaire, the Modern Health Worries scale, the Conspiracy Mentality Questionnaire, the 5C Scale variables, online media consumption behaviors, and vaccination behaviors and intentions for reliability and validity analyses. Alpha error level was set at 5%.

## Results

### Descriptive statistics

In study 1, data of 297 participants were included in the analyses (T1a), whereas 28 individuals participated at the second assessment four weeks after the first measurement date (T1b). The mean age of the sample of study 1 was 36.98 years (SD = 13.30), 74.07% of the participants were female. In the sample, 94.95% of the participants were from Germany. Higher education (with more than 13 years) was indicated in 59.26% of the participants, whereas 18.52% were students.

In the second study, 260 participants were included in the analyses (T2). The mean age of the sample was 34.92 years (SD = 14.40), 75.77% of the participants were female. In study 2, 97.31% of the participants were from Germany. Higher education was indicated in 64.62% of the participants, whereas 31.92% were students. The different mean and standard deviations in the assessed questionnaires for study 1 and study 2 are summarized in Table 1.

**Table 1:** Demographic characteristics and descriptive information from study 1 and study 2.

Sample characteristics	Study 1 (*N* = 297)	Study 2 (*N* = 260)	MD	***p***
Age [years], *M* (SD)	36.98 (13.30)	34.92 (14.40)	*t*(555) = 2.06	0.079
Gender [f / m / d]^a^, *n* (%)	220 (74.1)/ 74 (24.9)/3 (1.0)	197 (75.8)/ 61 (23.5)/2 (0.8)	*χ*^*2*^(2) = 0.26	0.876
Health occupation, *n* (%)	79 (26.6)	60 (23.1)	*χ*^*2*^(1) = 0.92	0.377
Self-rated health^b^, *M* (SD)	4.10 (0.76)	3.96 (0.72)	*t*(555) = 0.14	0.024
Current influenza vaccination status, *n* (%)	72 (24.2)	54 (20.8)	*χ*^*2*^(1) = 0.96	0.361
> 13 years of education, *n* (%)	176 (59.26)	168 (64.62)	*χ*^*2*^(1) = 1.68	0.221
Antivaccination attitudes (VAX), *M* (SD)	2.80 (1.25)	2.89 (1.02)	*t*(553) = −0.09	0.327
Perceived sensitivity to medicine, *M* (SD)	9.56 (4.65)	11.37 (4.43)	*t*(555) = −1.81	< 0.001
Beliefs about medicine, *M* (SD)	2.81 (0.79)	2.72 (0.69)	*t*(555) = 0.09	0.149
Modern health worries, *M* (SD)	2.70 (0.80)	2.59 (0.73)	*t*(554) = 0.10	0.105
Conspiracy mentality, *M* (SD)	5.56 (2.52)	4.46 (2.27)	*t*(555) = 1.10	< 0.001
5C complacency, *M* (SD)	2.62 (1.39)	2.49 (1.19)	*t*(555) = 0.13	0.238
5C calculation, *M* (SD)	5.16 (1.29)	5.13 (1.23)	*t*(555) = 0.03	0.775
5C confidence, *M* (SD)	4.88 (1.59)	4.88 (1.37)	*t*(555) = 0.01	0.961
5C collective responsibility, *M* (SD)	5.75 (1.37)	5.68 (1.27)	*t*(555) = 0.08	0.491
Online media consumption, *M* (SD)	3.98 (1.23)	4.24 (1.22)	*t*(555) = −0.26	0.014

*M*, mean; *SD*, standard deviation*.* a Gender [female / male / diverse]. b Self-rated health assessed by the item “How would you describe your current state of health?” based on the questionnaire SF-36 Health Survey (Morfeld & Bullinger, 2008) on a six-point Likert-type scale ranging from 1 ‘very poor’ to 5 ‘very good’.

### Confirmatory factor analysis (1)

The confirmatory factor analysis (CFA) was conducted on two samples, both based on a four-factor structure.

Sample 1: The results suggest excellent fit indices, with a Comparative Fit Index (CFI) of 0.991, a good Normed Fit Index (NFI) of 0.978, a very good Tucker-Lewis Index (TLI) of 0.988, and an excellent Root Mean Square Error of Approximation (RMSEA) of 0.048 (90% confidence interval: 0.029 – 0.066). Factor loadings range from 0.80 to 0.96, indicating strong associations between the items and their respective latent factors.

**Fig. 2:**
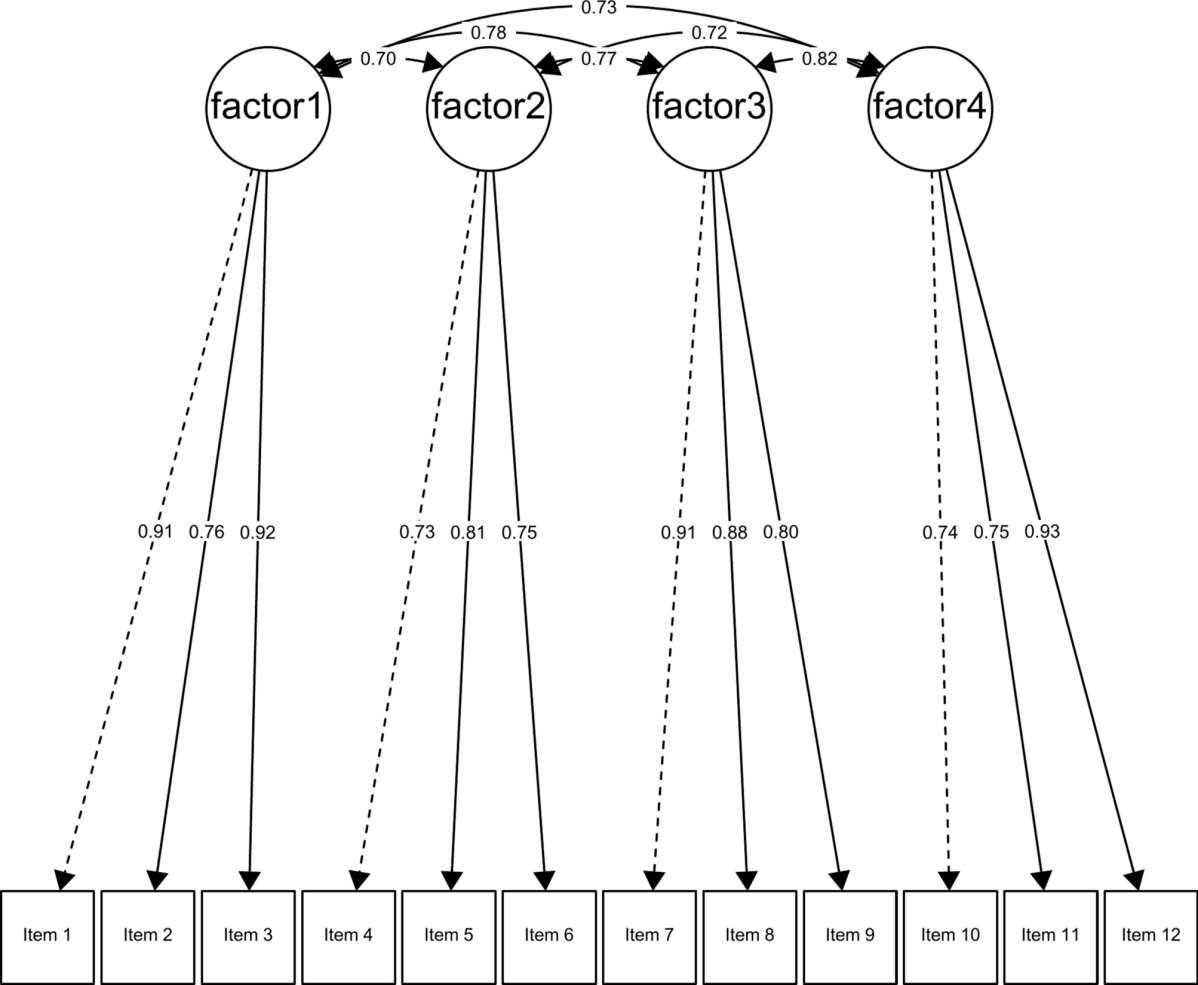
Path diagram and standardized estimated parameters of the CFA of the VAX scale (Sample 2). Factor (1) Mistrust of vaccine benefit, (2) Worries about unforeseen future effects, (3) Concerns about commercial profiteering, and (4) Preference for natural immunity.

Sample 2: The results for Sample 2 also suggest excellent fit indices, with a Comparative Fit Index (CFI) of 0.996, a very good Tucker-Lewis Index (TLI) of 0.994, and a Normed Fit Index (NFI) of 0.975. The Root Mean Square Error of Approximation (RMSEA) is 0.028, with a 90% confidence interval ranging from 0.000 to 0.051. Factor loadings for Sample 2 range from 0.73 to 0.93 (see Fig. 2), still demonstrating strong associations between the items and their respective latent factors. These indices further confirm the strong fit of the four-factor model to the data in Sample 2.

### Reliability analyses (2)

In study 1 and study 2, all items showed high item-total correlations between 0.59 and 0.86 with no value below 0.40^33^. The VAX scale and its proposed subscales showed excellent internal consistency in both studies with Cronbach´s alpha study 1 α_study 1_ = 0.96; Cronbach´s alpha study 2 α_study 2_ = 0.93 (Table 2). Furthermore, a high retest-reliability in study 1 was found with *r*_*tt*_ = 0.87 (*t*(26) = 8.84, *p* < 0.001). The retest-reliability between study 1 and study 2 (T1-T2) was similarly high with *r*_*tt*_ = 0.87 (*t*(24) = 8.48 , *p* < 0.001).

**Table 2:** Item analyses of study 1 and study 2 and Alpha reliabilities, Means, and standard deviations for VAX scale and its proposed subscales.

	Study 1			Study 2		
	Item difficulty	Item-total correlation	Variance	Item difficulty	Item-total correlation	Variance
Item 1	0.52	0.85	2.87	0.52	0.75	2.55
Item 2	0.55	0.75	2.79	0.54	0.69	2.46
Item 3	0.51	0.85	2.84	0.50	0.77	2.48
Item 4	0.72	0.72	2.93	0.80	0.59	2.44
Item 5	0.65	0.81	2.88	0.68	0.68	2.63
Item 6	0.58	0.84	3.13	0.73	0.64	2.95
Item 7	0.51	0.85	2.95	0.52	0.82	2.71
Item 8	0.52	0.84	3.05	0.49	0.80	2.77
Item 9	0.42	0.85	2.99	0.42	0.70	2.71
Item 10	0.63	0.76	3.16	0.66	0.65	2.92
Item 11	0.59	0.77	2.95	0.58	0.65	2.67
Item 12	0.50	0.86	2.97	0.52	0.80	2.76

	Study 1			Study 2		
	Mean	SD	Cronbach’s α	Mean	SD	Cronbach’s α
Vaccination Attitudes Examination Scale	2.80	1.25	.96	2.89	1.02	.93
Trust/mistrust in vaccine benefit	2.64	1.33	.94	2.61	1.13	.90
Worries over unforeseen future effects	3.26	1.36	.90	3.68	1.14	.80
Concerns about commercial profiteering	2.43	1.39	.92	2.37	1.25	.90
Preference for natural immunity	2.87	1.37	.89	2.92	1.22	.85

*SD*, standard deviation.

### Construct validity analyses (3)

The VAX scale values showed consistent correlations across both studies, supporting its construct validity. Strong positive correlations were observed with negative beliefs about medicine, conspiracy mentality, and modern health worries in both Study 1 (*r*_T1_ values from 0.57 to 0.76, all *p* < 0.001) and Study 2 (*r*_T2_ values from 0.42 to 0.68, all *p* < 0.001). Similarly, positive associations were found with the 5C sub-dimensions of complacency, calculation, and constraints in both studies (*r*_T1_ = 0.12 to 0.79; *r*_T2_ = 0.13 to 0.70, *p* < 0.05). Notably, strong negative correlations emerged between the VAX values and the 5C confidence and collective responsibility subscales in both samples (*r*_T1_ = −0.90 and −0.82; *r*_T2_ = −0.90 and −0.79, both *p* < 0.001). No significant correlation was found between VAX values and online media consumption behavior in either study (both *p* > 0.05). Full details of these correlations are presented in Table 3.

**Table 3:** Correlation matrix showing interrelationships between mean VAX scale value and means of relevant variables.

Measure	VAX	PSM	BMQ	MHW	CMQ	5C_**complacency**_	5C_**calculation**_	5C_**constraints**_	5C_**confidence**_	5C_collective responsibility_
VAX	-									
PSM		-								
Study 1	.53**									
Study 2	.43**									
BMQ			-							
Study 1	.76**	.49**								
Study 2	.68**	.47**								
MHW				-						
Study 1	.57**	.43**	.62**							
Study 2	.42**	.27**	.45**							
CMQ					-					
Study 1	.60**	.31**	.57**	.51**						
Study 2	.62**	.24**	.50**	.42**						
5C_complacency_						-				
Study 1	.79**	.48**	.64**	.47**	.46**					
Study 2	.70**	.28**	.54**	.31**	.42**					
5C_calculation_							-			
Study 1	.40**	.29**	.33**	.31**	.21**	.35**				
Study 2	.45**	.25**	.38**	.36**	.25**	.35**				
5C_constraints_								-		
Study 1	.12*	.26**	.17**	.02	.05	.22**	.07			
Study 2	.13*	.18**	.15*	.02	.14*	.20**	.06			
5C_confidence_									-	
Study 1	-.91**	-.47**	-.70**	-.50**	-.55**	-.75**	-.37**	-.08		
Study 2	-.90**	-.41**	-.62**	-.40**	-.57**	-.67**	-.41**	-.16*		
5C_collective responsibility_										-
Study 1	-.82**	-.43**	-.64**	-.49**	-.49**	-.78**	-.31**	-.19**	.84**	
Study 2	-.79**	-.38**	-.58**	-.34**	-.51**	-.69**	-.34**	-.17**	.81**	
Online media consumption										
Study 1	-.04	-.05	-.05	-.13*	-.02	-.04	-.02	-.09	.00	.04
Study 2	.01	-.05	.01	.01	.01	-.01	.00	-.03	-.02	.01

Two asterisks [**] indicate *p* < .01, one asterisk indicates *p* < .05 (two-tailed tested); *VAX*, Vaccination Attitudes Examination Scale. *PSM*, Perceived Sensitivity to Medicine Scale. *BMQ*, Beliefs About Medicines Questionnaire. *MHW*, Modern Health Worries Scale. *CMQ*, Conspiracy Mentality Questionnaire. *5C*, Psychological Antecedents of Vaccination Behavior Scale subdimensions.

### Criterion validity analyses (4)

**Fig. 3:**
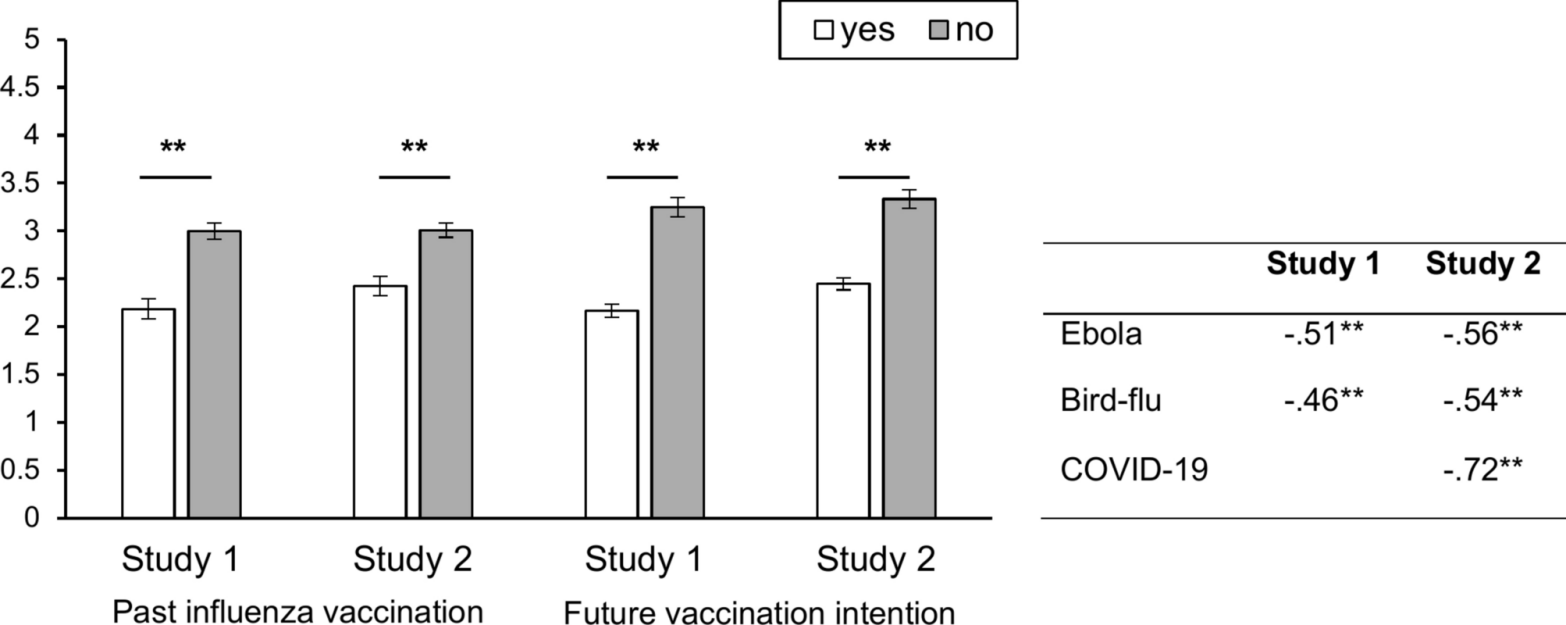
Criterion validity measures: Mean VAX scale values, vaccination behavior, and intentions. Error bars are ± 1 SE; asterisks [**] indicate *p* < 0.001, two-tailed tested.

VAX scale scores were found to be significantly higher in individuals who had not been previously vaccinated against influenza in both study 1 (*M*_yes_ = 2.18, SD = 0.90, *M*_no_ = 3.00, SD = 1.28; *t*(170) = −5.96, *p* < 0.001, *d* = −0.68) and study 2 (*M*_yes_ = 2.42, SD = 0.74, *M*_no_ = 3.01, SD = 1.06; *t*(117) = −4.64, *p* < 0.001, *d* = −0.58). The same applied to future intentions to be vaccinated with influenza in both study 1 (*M*_yes_ = 2.17, SD = 0.76, *M*_no_ = 3.25, SD = 1.33; *t*(282) = −8.86, *p* < 0.001, *d* = −0.96) and study 2 (*M*_yes_ = 2.45, SD = 0.72, *M*_no_ = 3.33, SD = 1.09; *t*(226) = −7.77, *p* < 0.001, *d* = −0.96). In addition, significant negative correlations between the average VAX scale score and the vaccine readiness for Ebola (*r*_T1_ = −0.51, *t*(295) = −10.05, *p* < 0.001; *r*_T2_ = −0.56, *t*(258) = −10.81,* p* < 0.001) and avian flu (*r*_T1_ = −0.46, *t*(295) = −8.93, *p* < 0.001; *r*_T2_ = −0.54, *t*(258) = −10.18,* p* < 0.001) were found. In Study 2, there was a highly significant association between future vaccination intentions against COVID-19 and the average VAX scale value (*r*_T2_ = −0.72, *t*(258) = −16.69,* p* < 0.001). Since only 26 respondents could be identified who had already taken part in study 1, a longitudinally evaluation of the T1-T2 data may not have been meaningful. A high average VAX value at T1 though was highly correlated with low COVID-19 vaccination intentions at T2 (*r*_T1-T2_ = −0.77, *t*(24) = −5.87,* p* < 0.001). Figure 3 summarizes all results and shows the *t*-test results in the bar graph as well as the bivariate associations between the average VAX scale values and the probability of being vaccinated against the mentioned viruses.

## Discussion

The presented scale is the first German translation of the Vaccination Attitude Examination Scale as developed by Martin and Petrie^8^, which makes it applicable in German-speaking countries. In two studies its psychometric properties and factor structure was examined in two non-restricted, predominantly German samples. The first survey was conducted before the COVID-19 pandemic broke out nationwide and restrictions on social life began. A second survey was carried out during the pandemic, but before an effective vaccine was approved. Overall, (a) the CFA performed in both samples confirmed the proposed four-factor structure of the twelve-item scale with very good to excellent fit measures. (b) The translated VAX scale demonstrated high retest-reliability and internal consistency estimates. (c) Convergent and discriminant validity measures were generally high and as hypothesized except for online media consumption behavior. (d) Average VAX values significantly discriminated between persons priorly receiving or refusing an influenza vaccination as well as showed strong associations with expected vaccination behavior during a pandemic outbreak.

The CFAs performed in both samples confirmed the proposed four-factor structure of the twelve-item scale with very good fit measures. Alpha reliabilities and retest reliability estimates were similarly high as in the English version^8,15^. These results suggest that the German translation of the VAX scale reliably measures the underlying construct of anti-vaccination attitudes.

Validity analyses indicated significant correlations between average VAX scale values and related constructs according to the hypotheses assumed a priori. Strong relationships were found in particular between negative beliefs about medicines and the 5C subscales complacency, confidence and collective responsibility. Correlations with the Beliefs about Medicine Questionnaire were even higher than in other studies examining COVID-19 specific concerns^34^. The resulted strong association of the average VAX values and the BMQ means is consistent with the necessity-concern framework^16^. Developed based on various theories, including Ajzen’s theory of planned behavior^35^, this framework posits the influence of behavioral beliefs in medicine-use patterns^24^. Accordingly, beliefs about medicine were shown to be stronger predictors of medication adherence than, for example, clinical or sociodemographic variables^16,25^.

Similarly, the conceptualization of the 5C subscale confidence also relates to the theory of planned behavior^5,30^ It can even be assumed that this factor, as described by Betsch et al.^5^comes closest to anti-vaccination attitudes as measured by the VAX scale, even when coded inversely. Consistent with this assumption, the strongest negative correlation was found for the relation between the VAX scale and this 5C subscale. Complacency had a similarly high correlation as beliefs about medicine. In terms of the theory of planned behavior^35^, complacency could be understood as low accessibility to behavioral beliefs that might influence attitudes and vaccination intentions. The construct collective responsibility can rather be understood as a stable subjective value. This could explain the high correlation with the found anti-vaccination attitudes and its intercorrelations. However, it can also indicate that someone simply is unfamiliar with the concept of herd immunity^5^.

Items and sub-dimensions of the VAX scale are not based on any specific theoretical model, but were derived qualitatively on the basis of statements critical of vaccination^8^. Although a theoretical framework exists that attempts to explain and predict vaccine hesitancy and acceptance^3,5^, the approach of the VAX scale lends itself to uncover other facets of the fairly new construct. The correlations regarding conspiracy mentality were higher than in other studies (e.g., Betsch et al.^5^). The reason for this is probably its qualitative development, which also contains, for example, critical statements of vaccination from relevant forums (e.g., “Vaccination programs are a big con”). However, this may mean that the inclusion of such statements better operationalizes the construct of anti-vaccination attitudes.

In line with the theory of planned behavior^35^, there is a link between average VAX values, past and intended vaccination behavior^36^. Significant differences were found between individuals who had been vaccinated against influenza in the past season and those who had not, as well as those who intended to be vaccinated next season and vice versa. In addition, moderate to strong correlations were found between average VAX values and the probabilities that participants would be vaccinated if exposed to a pandemic. Unfortunately, the present results only include correlations. Since only 26 participants who participated in both studies were identified, a regression analysis would not have been adequate. What is striking though is a high test–retest reliability found when assessing the T1-T2 sample and a strong correlation between the average VAX scale values and the intention to receive a vaccination against COVID-19 at T2. This suggests that the German translation of the Vaccination Attitude Examination Scale is a useful tool for measuring this rather stable construct of anti-vaccination attitudes. In addition, it can be assumed that nothing fundamental has changed in the VAX factors surveyed due to the influence of the pandemic, so that the present scale captures anti-vaccination attitudes regardless of the circumstances of the pandemic.

### Research and practical implications

Martin and Petrie^8^developed the scale on the assumption that anti-vaccination attitudes are not vaccine-specific, thus contradicting the common definition of vaccine hesitancy^3^. It is therefore necessary to examine whether individual vaccination attitudes are robust across different vaccines. Since the VAX scale is also available in other languages^11,12^, anti-vaccination attitudes and associated cognitions and behaviors can be compared across national borders, and cultural differences or similarities can be examined. In a next step, future research can conduct longitudinal and experimental studies that record the link between vaccination attitudes and actual vaccination behavior. First studies already showed connections between the VAX scale and the rejection of vaccinations even after 12 months^37^. Based on the theory of planned behavior^35^, this approach can also shed light on the association between unpleasant previous vaccination experiences, the availability of behavioral beliefs, and the development of anti-vaccination attitudes. Better recording and understanding of anti-vaccine attitudes can be used to evolve tailored interventions to address vaccine hesitancy^8,10^. One approach that has already been studied to counter vaccine hesitancy is the use of communication frames, but these have been shown to have varying degrees of effectiveness^38^. Anti-vaccination attitudes measured with the translated VAX scale moderated the effects of expectation-optimizing verbal information on vaccination uptake willingness^39^. People with high anti-vaccination attitudes tended to show even lower vaccination intentions after a vaccination information was positively framed. A better understanding of the development of anti-vaccination attitudes may therefore help to identify influences that can promote negative attitudes at an early stage and to counteract them as early as possible. A valid instrument for measuring anti-vaccination attitudes such as the VAX scale opens the possibility to develop tailored communications frames to target attitude-based vaccine hesitancy.

### Strengths and limitations

The present sample is a non-probabilistic opportunity sample that is subject to a possible self-selection bias. At the beginning of the study, participants were informed that they would be asked questions about their vaccination attitudes and vaccination behavior, which may have attracted individuals with particularly strong views (either positive or negative) on vaccination, especially in a politically charged climate during the pandemic. The timing of data collection may partially explain the differences observed between samples and the varying results of the factor analyses. Additionally, as noted, the results are correlational and do not allow for causal interpretations. Another limitation is the demographic composition of the sample, which was predominantly younger, female, highly educated, and from a single country. These characteristics may affect the generalizability of the findings, as vaccination attitudes could vary across different demographic groups and cultural contexts. Future studies should aim for more diverse sampling to strengthen the external validity of the VAX Scale. This study also has several strengths: By validating the questionnaire with two large population-based samples before and during the global COVID-19 pandemic, the German translation of the VAX Scale provides meaningful results. The good psychometric properties of the instrument suggest that it reliably and validly captures anti-vaccination attitudes, regardless of an ongoing pandemic.

## Conclusion

The German translation of the Vaccination Attitudes Examination (VAX) Scale is a reliable and valid measure of anti-vaccination attitudes in German-speaking populations. The robust four-factor structure remained consistent across pre-pandemic and pandemic samples, demonstrating strong internal consistency and test–retest reliability. The scale effectively distinguishes between vaccination behaviors, making it a valuable tool for cross-cultural comparisons and targeted interventions against vaccine hesitancy.

This consistency highlights the scale’s utility in understanding stable anti-vaccination attitudes over time, offering crucial insights for healthcare providers to develop tailored strategies that enhance vaccine uptake, especially in response to public health crises like COVID-19.

## Electronic Supplementary Material

Below is the link to the electronic supplementary material.

Supplementary Material 1

## Data Availability

All data and codes used for the analyses described will be made available upon reasonable request from the corresponding author.
