# Supplementary material for: Psychometric validation of the Vaccination Attitudes Examination (VAX) scale in German pre-pandemic and mid-pandemic samples
Source: Sci Rep. 2024 Dec 18;14:30543. doi: 10.1038/s41598-024-82726-8 (PMC11655520; doi:10.1038/s41598-024-82726-8)
Supplement: Supplementary file 1 — Supplementary Material 1 [file 41598_2024_82726_MOESM1_ESM.docx]

**Supplement** Original items and translation

| English | German |
| --- | --- |
| 1. I feel safe after being vaccinated | Ich fühle mich sicher, nachdem ich geimpft wurde. |
| 2. I can rely on vaccines to stop serious  infectious diseases | Ich kann mich darauf verlassen, dass Impfstoffe schwere Infektionskrankheiten abwehren. |
| 3. I feel protected after getting vaccinated | Ich fühle mich geschützt, nachdem ich geimpft wurde. |
| 4. Although most vaccines appear to be safe, there may be problems that we have not yet discovered | Auch wenn die meisten Impfstoffe sicher zu sein scheinen, gibt es vielleicht Probleme, die wir noch nicht entdeckt haben. |
| 5. Vaccines can cause unforeseen problems in  children | Impfstoffe können bei Kindern unvorhergesehene Probleme verursachen. |
| 6. I worry about the unknown effects of  vaccines in the future | Ich mache mir Sorgen über unbekannte Auswirkungen von Impfstoffen in der Zukunft. |
| 7. Vaccines make a lot of money for pharmaceutical companies, but do not do much for regular people. | Impfstoffe bringen Pharma-Unternehmen eine Menge Geld ein, sie nutzen den Menschen jedoch nicht viel. |
| 8. Authorities promote vaccination for financial gain, not for people’s health | Die Behörden fördern Impfungen aus wirtschaftlichem Nutzen, nicht wegen der Gesundheit der Menschen. |
| 9. Vaccination programs are a big con | Impfprogramme sind ein großer Betrug. |
| 10. Natural immunity lasts longer than a vaccination | Eine natürliche Immunität hält länger als eine Impfung. |
| 11. Natural exposure to viruses and germs gives the safest protection. | Natürlicher Kontakt mit Viren und Keimen gibt den sichersten Schutz. |
| 12. Being exposed to diseases naturally is safer for the immune system than being exposed through vaccination | Es ist für das Immunsystem sicherer, Krankheiten natürlich ausgesetzt zu sein als durch eine Impfung. |
